# The Dangers of Barbecuing: An Interesting Case of a Foreign Body in the Throat

**DOI:** 10.5811/cpcem.2019.4.42105

**Published:** 2019-05-29

**Authors:** Vincent R. Sicari, Joseph Pepe, Alfonso C. Cardenas, Christopher Zabbo

**Affiliations:** Kent Hospital, Department of Emergency Medicine, Warwick, Rhode Island

## Abstract

Swallowing of foreign bodies (FB), and sensation of such in the throat, is a common complaint in the emergency department setting, with roughly 80,000 visits in 2010 for FB ingestion.^1^ Grill wire brushes are a rarely reported, accidental FB ingestion, although recent literature suggests that it is more common than initially thought.^2^ This is a report of a female with acute onset odynophagia after a meal, with a normal laryngoscopic exam that used flexible fiberoptics. Evidence of a metallic linear density was present in the retropharynx on computed tomography imaging, most consistent with a wire from a grill wire brush.

## CASE PRESENTATION

**Image 1:**
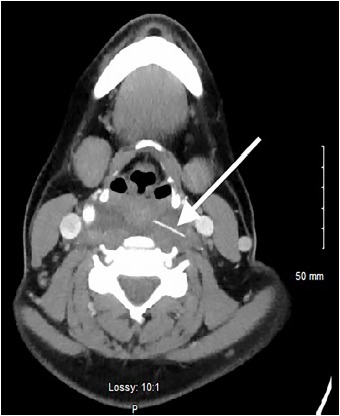
Computed tomography of the neck soft tissue with intravenous contrast in axial view, showing a linear metallic density with surrounding retropharyngeal effusion (white arrow).

**Image 2:**
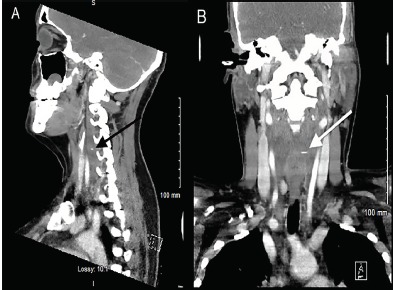
Computed tomography of the neck soft tissue with intravenous contrast in sagittal (A) and coronal (B) views. A linear metallic density with surrounding retropharyngeal effusion is visible: (A) (black arrow) and (B) (white arrow).

A 36-year-old-female presented to the emergency department with a one-day history of sharp pain in her throat that began immediately after swallowing food. Her vital signs, chest radiograph, and physical exam were unremarkable. She was discharged home after flexible fiberoptic visualization of her posterior oropharynx was normal. Her symptoms had subsided after treatment with viscous lidocaine. After she returned four days later with odynophagia, neck computed tomography (CT) was performed that showed a two-centimeter linear, radiopaque metallic density within the retropharyngeal soft tissues with effusion (Images 1 and 2).

## DISCUSSION

The patient was diagnosed with a retropharyngeal effusion caused by a retained foreign body (FB). She was taken to the operating room by otolaryngology; however, an attempt at retrieval was unsuccessful. She was subsequently transferred to a facility with head and neck surgery for the retrieval. On her first day of symptoms she did recall using a grill-wire brush prior to placing the food she had eaten on the grill, and therefore the FB was thought to be a wire from the brush. Grill wire brushes are a rarely reported, accidental FB ingestion, although recent literature suggests that it is more common than once thought.^1^ Recently there have been increasing, albeit few, case reports describing grill-wire brush ingestion and their potential complications, which include esophageal perforation, effusion, abscess, and death.^2^,^3^,^4^ This case demonstrates that patients with a clinical history suggestive of grill-wire brush FB should be considered for CT imaging, even in the absence of other physical findings.

**CPC-EM Capsule**

What do we already know about this clinical entity?

Foreign body (FB) sensation in the throat is typically a benign condition. Grill wire brush ingestions are a rarely reported accidental FB ingestion but more common than once thought.

What is the major impact of the image(s)?

An accurate patient history and a high index of suspicion for this rare ingestion will decrease the delay in diagnosing this potentially life-threatening condition.

How might this improve emergency medicine practice?

Probing further into patient history, and adding contrast-enhanced imaging to plain radiographs, would help delineate this condition and its associated complications.
